# Genome-wide profiling of histone H3 lysine 27 and lysine 4 trimethylation in multiple myeloma reveals the importance of Polycomb gene targeting and highlights EZH2 as a potential therapeutic target

**DOI:** 10.18632/oncotarget.6843

**Published:** 2016-01-07

**Authors:** Prasoon Agarwal, Mohammad Alzrigat, Alba Atienza Párraga, Stefan Enroth, Umashankar Singh, Johanna Ungerstedt, Anders Österborg, Peter J. Brown, Anqi Ma, Jian Jin, Kenneth Nilsson, Fredrik Öberg, Antonia Kalushkova, Helena Jernberg-Wiklund

**Affiliations:** ^1^ Science for Life Laboratory, Department of Immunology, Genetics and Pathology, Rudbeck Laboratory, Uppsala University, Uppsala, Sweden; ^2^ Department of Biological Engineering, Indian Institute of Technology, Gandhinagar, Gujarat, India; ^3^ Department of Medicine, Center for Hematology and Regenerative Medicine (HERM), Karolinska Institute Huddinge, Stockholm, Sweden; ^4^ Department of Oncology-Pathology, Karolinska University Hospital Solna, Stockholm, Sweden; ^5^ Structural Genomics Consortium, University of Toronto, Toronto, Ontario, Canada; ^6^ Departments of Structural and Chemical Biology, Oncological Sciences, and Pharmacology and Systems Therapeutics, Icahn School of Medicine at Mount Sinai, New York, NY, USA

**Keywords:** multiple myeloma, Polycomb, EZH2, H3K27me3, UNC1999

## Abstract

Multiple myeloma (MM) is a malignancy of the antibody-producing plasma cells. MM is a highly heterogeneous disease, which has hampered the identification of a common underlying mechanism for disease establishment as well as the development of targeted therapy. Here we present the first genome-wide profiling of histone H3 lysine 27 and lysine 4 trimethylation in MM patient samples, defining a common set of active H3K4me3-enriched genes and silent genes marked by H3K27me3 (H3K27me3 alone or bivalent) unique to primary MM cells, when compared to normal bone marrow plasma cells. Using this epigenome profile, we found increased silencing of H3K27me3 targets in MM patients at advanced stages of the disease, and the expression pattern of H3K27me3-marked genes correlated with poor patient survival. We also demonstrated that pharmacological inhibition of EZH2 had anti-myeloma effects in both MM cell lines and CD138+ MM patient cells. In addition, EZH2 inhibition decreased the global H3K27 methylation and induced apoptosis. Taken together, these data suggest an important role for the Polycomb repressive complex 2 (PRC2) in MM, and highlights the PRC2 component EZH2 as a potential therapeutic target in MM.

## INTRODUCTION

Multiple myeloma (MM) is a plasma cell tumor localized to the bone marrow. The disease is further characterized by lytic bone lesions and immunodeficiency associated with monoclonal protein in the blood and/or urine [1, 2]. The myeloma cell resembles a post-germinal center, isotype-switched, long-living plasma cell with retained proliferation potential [3]. According to the current view, MM is a genetically complex and heterogeneous disease characterized by the accumulation of several genetic lesions, such as numeric and structural chromosomal aberrations, and gene mutations in different pathways [2]. Recent sequencing projects indicate the absence of common underlying genetic events as drivers of MM pathogenesis and underline the large intra- and interpatient heterogeneity that may limit the clinical benefits of targeted therapy [4–8].

In addition to genetic abnormalities, recent studies have shown that deregulation of epigenetic mechanisms could play an essential role in MM development [9]. The t(4;14) translocation affects 15% of patients and is considered a marker of poor prognosis in MM [10]. The t(4;14) translocation highlights the importance of epigenetic modulators in MM, as the multiple myeloma SET domain (MMSET) is known to establish dimethylation of histone H3 at lysine 36 (H3K36me2) [11]. Recently, the overexpression of MMSET in MM has been shown to alter the global chromatin landscape i.e. increased H3K36me2 and redistribution of H3K27me3 [12, 13]. Additionally, in a proportion of primary MM and MM cell lines lacking t(4;14) a recurrent mutation in the H3K27 demethylase KDM6A (also known as UTX) has been reported [14], further highlighting an emerging role of chromatin modifiers in MM. In these cases, however, epigenetic deregulation has so far only been observed in a subpopulation of patients. When focusing on the common underexpressed genes in MM plasma cells as compared with the normal counterpart we have previously identified a set of genes targeted by the Polycomb repressive complex 2 (PRC2) [15]. PRC2 is a transcriptional repressor with an important role during development and differentiation [16]. Its catalytic subunits, enhancer of zeste homologue 2 (EZH2) or the related EZH1, trimethylate lysine 27 on histone H3 which is associated with gene silencing and monoubiquitination of histone H2A [17] and/or DNA-methylation [18, 19]. Pathologic activation of EZH2 by genetic alterations has been documented in various cancers, including hematological tumors, making EZH2 a potential therapeutic target [20, 21]. In MM, EZH2 has previously been suggested to have oncogenic properties based on its overexpression and correlation of histone methyltransferase activity to tumor formation *in vivo* [22].

In this study, the aim was to investigate the genome-wide distribution of H3K27me3 and H3K4me3 in MM and to test whether pharmacological inhibition of the methyltransferase activity of EZH2 would demonstrate anti-myeloma potential. Using ChIP-sequencing, we define MM-unique Polycomb (H3K27me3 alone and bivalent) target genes when compared with targets of this complex defined in normal bone marrow plasma cells. We show that the MM-unique H3K27me3-enriched genes significantly overlap with underexpressed genes in MM patients in ISS stage III, as compared with stage I and II, and in patients with poor survival in independent clinical MM studies. We further illustrate that two selective small chemical EZH2 inhibitors, UNC1999 and GSK343, reduced the survival of MM cell lines and primary cells. EZH2 inhibition decreased H3K27 methylation marks, induced apoptosis and inhibited colony formation in MM cell lines. These data strengthen our previous hypothesis on Polycomb involvement in MM and highlight EZH2 as a promising therapeutic target in MM.

## RESULTS

### Chromatin profiles and transcriptional states in MM patient samples

Using an integrative genomic approach, we recently provided proof-of-principle that gene silencing associated with H3K27me3 was increased in malignant MM cells compared to their normal counterparts [15]. We therefore hypothesized that Polycomb gene targeting may contribute to MM tumorigenesis. In this study, we sought to investigate the genome-wide distribution of H3K27me3 and H3K4me3 marks in MM by performing ChIP-sequencing on freshly isolated CD138^+^-sorted cells from four newly diagnosed MM patients (Figure S1 and Table S1). At time of sampling, characterization by FISH analysis was not part of clinical routine at the sample collection center, thus MM patient stratification other than according to the international staging system (ISS) was not available. Subsequent FISH analysis was performed on patient whole bone marrow smear samples collected at diagnosis but did not produce a positive signal for the most common chromosomal abnormalities observed in MM including t(4;14), t(11;14), t(14;16), t(8;14) and +8 (data not shown).

**Figure 1:**
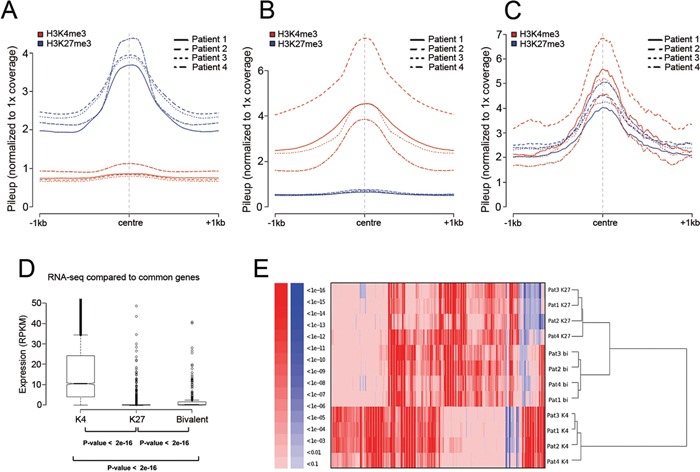
The chromatin profile and transcriptional activity of MM **A-C.** Normalized pileup signals after peak calling plotted along the center of the peaks for all the four patients. Blue color represents H3K27me3 and red represents H3K4me3 in all panels. X-axis presents 1000 bp up- and downstream from the center of the peak. Y-axis presents raw reads normalized to genome coverage of 1X. **A.** Regions enriched for H3K27me3 and lacking H3K4me3. **B.** Regions enriched for H3K4me3 and lacking H3K27me3. **C.** Regions with presence of both H3K27me3 and H3K4me3. **D.** Correlation between presence of the chromatin marks H3K27me3 and H3K4me3 defined by ChIP-sequencing and gene expression defined by RNA-sequencing. Genes in each enriched category after ChIP-seq were plotted against RPKM values representing gene expression (Y-axis) from RNA-seq. P-values were calculated using Mann-Whitney test (p <10e-16). **E.** Hierarchical clustering of gene ontology (GO) categories of biological processes for the genes defined as enriched for H3K27me3 (K27), H3K4me3 (K4) or bivalent genes enriched for both (bi) as defined for each patient by ChIP-seq. Clustering was performed on P-values of over-(red) and under-(blue) represented GO categories.

We then generated enriched regions with H3K27me3, H3K4me3, or with both marks present (bivalent) for the four MM patient samples (Figure 1A-1C). Based on the selection criteria of mark enrichment we compiled lists of 1205 targets of H3K27me3 common among the four patients, 5269 common genes enriched for H3K4me3, and 281 common bivalent genes (Table S5). The corresponding number of peaks assigned for each chromatin profile in each patient and their contribution to the common lists are outlined in Table S6.

To correlate the presence of a defined chromatin mark to transcriptional activity we compared the enrichment for each chromatin mark among all patients to data obtained by RNA-sequencing from CD138^+^ cells derived from patient 3 (Figure 1D). H3K27me3 commonly marks silenced genes [23, 24] while H3K4me3 is a hallmark of actively transcribed genes [25]. We found both H3K27me3 and bivalent genes to have significantly lower expression than H3K4me3 targets. Bivalent genes showed significantly higher expression when compared with H3K27me3 targets. To gain a deeper knowledge of the genes defined by each chromatin mark and how they functionally relate to each other, we performed hierarchical clustering based on functional annotation of the genes as defined for each patient (Figure 1E). Notably, the bivalent genes shared a similar profile with genes enriched for H3K27me3 alone forming one cluster with similar functional groups, while the H3K4me3-marked genes formed a separate cluster.

### The MM-unique H3K27me3-enriched genes overlap with previously defined Polycomb targets and underexpressed genes in large independent MM cohort studies

**Figure 2:**
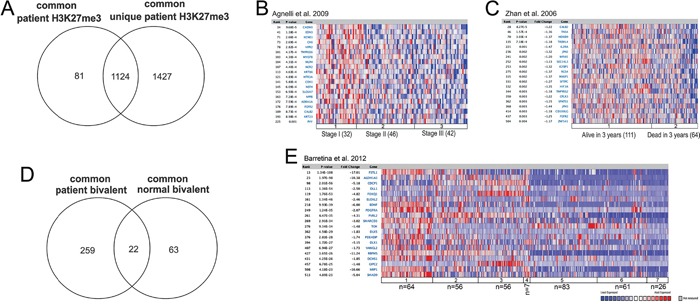
The MM H3K27me3 and bivalent genes overlap with previously defined Polycomb targets and gene repression in MM patients **A.** Defining the MM myeloma common and unique H3K27me3. The 1205 MM H3K27me3 targets common among the 4 patients were intersected with the 2551 MM unique H3K27me3 targets defined when using H3K27me3 enrichment in normal plasma cells as a background to identify 1124 common and unique MM H3K27me3 targets. Intersection was generated by using Venny 2.0.2. [67] **B-C.** Heatmap representation from gene expression studies identified through Oncomine including genes overlapping with the common and unique H3K27me3 targets as identified in this study. **B.** H3K27me3 targets are more silenced in MM ISS stage III, as compared with ISS stage I and II (Agnelli et al. 2009) and **C.** H3K27me3 targets are more silenced in MM patients presenting with poor survival (Zhan et al. 2006). **D.** Identification of 259 MM unique bivalent genes when compared to bivalent genes in normal bone marrow plasma cells. Intersection was generated by using Venny 2.0.2. [67] **E.** Heatmap representation of gene expression derived through Oncomine of the MM common and unique bivalent genes; gene expression in MM cell lines as compared with other cancer cell lines: 1- Brain and CNS Cancer (n=64), 2- Breast Cancer (n=56), 3- Colorectal Cancer (n=56), 4- Prostate Cancer (n=7), 5- Leukemia (n=83), 6- Lymphoma (n=61), 7- Myeloma (n=26).

To further investigate the H3K27me3-enriched genes in malignant MM cells, we included normal plasma cell samples derived from bone marrow of two age-matched healthy donors. Firstly, we performed ChIP-sequencing for H3K27me3 and H3K4me3 in the normal plasma cells and, by using the generated enrichment as background to the MM patient samples, we defined 2551 H3K27me3-enriched genes that were common to all MM patients used in this study and unique to MM when compared to normal bone marrow plasma cells. Secondly, we intersected these 2551 genes with the MM 1205 H3K27me3 targets defined in the first results section when patient data was analyzed alone to obtain a more stringent list of 1124 H3K27me3-enriched genes common to all patients and unique to MM when compared with normal bone marrow plasma cells (Figure 2A). Thirdly, to exclude the possibility that bivalent genes were present within the list of 1124 H3K27me3 targets, the bivalent genes defined for each patient were removed from this list. This resulted in 374 genes enriched for H3K27me3 only, which are common and unique to MM patients (Table S7). Subsequently, we examined the 374 H3K27me3 targets in MM for overlaps with previously-defined concepts in the Oncomine database [26], and found significant association to previously-defined Polycomb targets in human embryonic fibroblasts [27] and in human embryonic stem cells [28] (Table 1).

**Table 1:** Oncomine literature defined concepts for MM common and unique H3K27me3 targets

Literature defined concepts for MM H3K27me3 targets	P-value	Odds ratio	Overlap size	Q-value
Tri-methylated H3K27 target genes in human embryonic fibroblasts (Bracken et al. 2006)	1.60E-121	17.3	177	1.57E-116
CBX8 target genes in human embryonic fibroblasts (Bracken et al. 2006)	3.35E-61	8.6	127	1.65E-56
SUZ12 target genes in human embryonic fibroblasts (Bracken et al. 2006)	3.05E-47	10.9	77	9.98E-43
Tri-methylated H3K27 target genes in human embryonic stem cells (Lee et al. 2006)	3.05E-23	6.4	52	4.99E-19
SUZ12 target genes in human embryonic stem cells (Lee et al. 2006)	3.47E-22	6.5	49	4.86E-18
Polycomb Group target genes in human embryonic stem cells (Lee et al. 2006)	2.83E-15	6.5	32	2.53E-11
EED target genes in human embryonic fibroblasts (Bracken et al. 2006)	1.45E-13	4.7	38	8.88E-10

Odds ratio: represents how strongly the MM common and unique H3K27me3 targets belong to the corresponding data in Oncomine; Overlap size: represents the intersection of MM data generated in this study and the corresponding data in Oncomine; Q-value: represents the corrected p-value.

Furthermore, we compared the MM-unique H3K27me3-enriched genes to expression data from large and independent MM patient cohorts available through Oncomine. We found that the H3K27me3-enriched genes in MM significantly overlapped with underexpressed genes in ISS stage III as compared with ISS stage I and II [29] (Figure 2B and Table 2). In addition, the H3K27me3 genes were significantly enriched among underexpressed genes in plasma cell leukemia (PCL), representative of disseminated tumor at advanced stages of the MM disease, when compared to MGUS and MM together [29]. Notably, H3K27me3-enriched genes in MM significantly overlapped with genes underexpressed in MM patients with poor survival [30, 31] (Figure 2C and Table 2).

**Table 2:** Multiple myeloma studies correlating with the common and unique H3K27me3 targets

Myeloma studies correlating with H3K27me3 targets	P-value	Odds ratio	Overlap size	Q-value
Top 10% under-expressed in MM stage III vs I and II (Agnelli et al. 2009)	1.93E-12	3.4	57	9.45E-09
Top 10% over-expressed MGUS vs MM and PCL (Agnelli et al. 2009)	1.93E-12	3.4	57	9.95E-09
Top 10% under-expressed dead vs alive at 3 years (Zhan et al. 2006)	4.00E-09	2.4	67	6.33E-06
Top 10% under-expressed dead vs alive at 1 year (Mulligan et al. 2007)	1.74E-08	2.8	49	2.36E-05
Top 10% under-expressed in MM vs MGUS (Zhan et al. 2002)	2.61E-07	3.4	30	2.10E-04
Top 5% under-expressed in MGUS vs normal bone marrow (Zhan et al. 2007)	2.77E-07	2.7	39	2.16E-04
Top 10% under-expressed in PCL vs MM and MGUS (Agnelli et al. 2009)	2.19E-06	2.4	44	1.00E-03
Top 10% under-expressed in smoldering myeloma vs normal bone marrow (Zhan et al. 2007)	2.96E-06	2.1	59	1.00E-03

Odds ratio: represents how strongly the MM common and unique H3K27me3 targets belong to the corresponding data in Oncomine; Overlap size: represents the intersection of MM data generated in this study and the corresponding data in Oncomine; Q-value: represents the corrected p-value.

### Bivalent domains increase in MM as compared with normal plasma cells

The MM-common bivalent genes defined in this study were transcriptionally inactive and functionally resembled the H3K27me3 targets (Figure 1D and 1E). Interestingly, the number of bivalent genes increased in MM when compared with normal plasma cells, with a very small number of genes overlapping between these two entities (Figure 2D and Table S7). Similar to H3K27me3 targets, MM-unique bivalent genes were significantly enriched among previously-defined Polycomb targets in human embryonic fibroblasts [27] and human embryonic stem cells [28] (Table 3). However, in contrast to H3K27me3-enriched genes, bivalent genes further overlapped with genes found to be reactivated upon knockdown of Polycomb proteins [27] (Table 3). Furthermore, the MM-bivalent genes overlapped with genes underexpressed in MM cell lines when compared with other cancer cell lines of both solid and hematopoietic origin (Table 4, Figure 2E). Also, the MM-bivalent genes overlapped with the top 10% overexpressed genes in MM as compared with MGUS and PCL [32].

**Table 3:** Oncomine literature defined concepts for MM unique bivalent genes

Literature defined concepts for MM bivalent genes	P-value	Odds ratio	Overlap size	Q-value
Tri-methylated H3K27 target genes in human embryonic stem cells (Lee et al. 2006)	5.15E-74	21.9	88	5.05E-69
EED target genes in human embryonic fibroblasts (Bracken et al. 2006)	7.15E-72	21.9	85	3.51E-67
CBX8 target genes in human embryonic fibroblasts (Bracken et al. 2006)	3.82E-60	11.5	105	1.25E-55
SUZ12 target genes in human embryonic stem cells (Lee et al. 2006)	7.80E-56	17.4	72	1.91E-51
Polycomb Group target genes in human embryonic stem cells (Lee et al. 2006)	4.27E-48	20.3	56	8.38E-44
SUZ12 target genes in human embryonic fibroblasts (Bracken et al. 2006)	1.16E-42	13.3	62	1.90E-38
Tri-methylated H3K27 target genes in human embryonic fibroblasts (Bracken et al. 2006)	6.22E-38	8.1	79	8.72E-34
Activated upon Polycomb Group knockdown (Bracken et al. 2006)	2.78E-05	5.5	10	0.004

Odds ratio: represents how strongly the MM unique bivalent targets belong to the corresponding data in Oncomine; Overlap size: represents the intersection of MM data generated in this study and the corresponding data in Oncomine; Q-value: represents the corrected p-value.

**Table 4:** Multiple myeloma studies correlating with the bivalent targets

Myeloma studies correlating with bivalent genes	P-value	Odds ratio	Overlap size	Q-value
Top 10% under-expressed in MM cell line vs other cancer cell lines (Barretina et al. 2012)	7.04E-19	4.1	73	6.28E-15
Top 10% under-expressed in MM cell line vs other cancer cell lines (Wooster, unpublished GlaxoSmithKline)	7.04E-19	4.1	73	6.91E-15
Top 5% under-expressed in MM cell lines cell line vs other cancer cell lines (Garnett et al. 2012)	1.03E-11	4.7	34	2.51E-08
Top 10% over-expressed in MM vs MGUS and PCL (Chapman et al. 2011)	4.58E-06	2.2	47	9.48E-04

Odds ratio: represents how strongly the MM unique bivalent targets belong to the corresponding data in Oncomine; Overlap size: represents the intersection of MM data generated in this study and the corresponding data in Oncomine; Q-value: represents the corrected p-value.

### Pharmacological inhibition of EZH2 reduces viability, induces apoptosis and suppresses colony formation of MM cells *in vitro*

**Figure 3:**
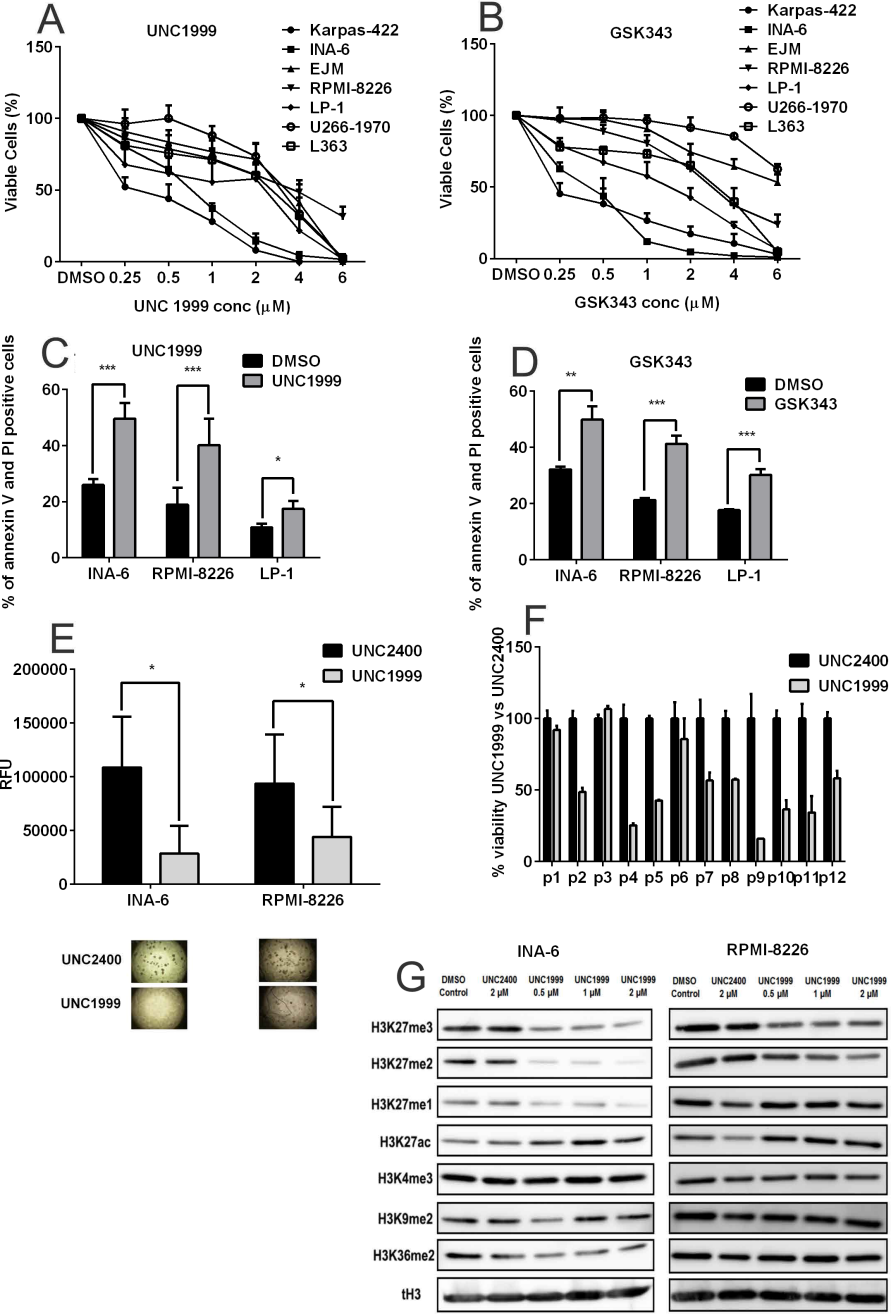
Pharmacological inhibition of EZH2 confers anti-myeloma effects **A.** UNC1999 and **B.** GSK343 reduced the viability of a panel of MM cell lines in a dose-dependent manner. Cells were treated with a range of concentrations and viability was analyzed at day 6 using the AlamarBlue assay. The Karpas-422 cell line was used as a control cell line. **C.** UNC1999 (2μM) and **D.** GSK343 (2μM) induced apoptosis in MM 72 hours posttreatment. DMSO was used as control treatment and apoptosis was measured by assessing percentage of Annexin V and PI positive cells using flow cytometry. **E.** Treatment with UNC1999 (2μM), but not with UNC2400, inhibited colony formation of the INA-6 and RPMI-8226 in methylcellulose media after 7 days. **F.** UNC1999 (4μM), but not UNC2400, reduced the viability of a majority of primary MM cells, as analyzed by the AlamarBlue assay at 72 hours posttreatment. **G.** Treatment with UNC1999 decreased H3K27me1, 2 and 3 methylation marks and led to an increase in H3K27ac mark in the INA-6 and RPMI-8226 cell lines. Other methylation marks remained unaffected. The MM cell lines were treated with a range of UNC1999 concentrations for 72 hours. DMSO and the inactive UNC2400 were used as controls. Total H3 is shown as loading control. The blots are representative of three independent biological experiments. All error bars represent standard deviation of at least three independent experiments except for (F) where error bars represent standard deviation of three technical replicates. P-values were calculated using two-tailed student t-test, p: *< 0.05; **<0.01; ***<0.001.

Based on the above, we hypothesized that Polycomb gene targeting may be of clinical relevance in MM. Thus, we further investigated whether inhibition of the EZH2 methyltransferase activity would demonstrate anti-myeloma effects *in vitro*. To explore this, we assessed the effect of the newly-developed specific EZH2 inhibitors UNC1999 [33] and GSK343 [34] on the viability of a panel of authenticated MM cell lines using the Alamar Blue assay. The human diffuse large B-cell lymphoma cell line Karpas-422, which harbors an EZH2 gain-of-function mutation (Y641N) and is sensitive to EZH2 inhibition [33, 35, 36], was used as a control. Ablation of EZH2 methyltransferase activity using both UNC1999 and GSK343 reduced the viability of MM cell lines in a dose- and time-dependent manner (Figure 3A and 3B and Figure S2). The viability of the MM cell line INA-6 was strongly suppressed by both inhibitors at a similar concentration range that has previously been demonstrated for Karpas-422 (Figure 3A and 3B). However, all MM cell lines included in this study harbored wild type EZH2 SET domain as identified by Sanger cDNA sequencing (data not shown).

Next, we investigated whether EZH2 inhibition induces apoptosis, cell cycle arrest and reduces colony formation in MM cells. As a control treatment we used either DMSO or the chemically inactive form of UNC1999, UNC2400 [33], which was found not to affect cell viability (Figure S3A). UNC1999 and GSK343 reduced MM cell viability by induction of apoptosis. Treatment with UNC1999 increased the number of apoptotic cells from 25% to 50% in the INA-6 cell line, from 19% to 40% in the RPMI-8226 cell line and from 10% to 18% in the LP-1 cell line (Figure 3C), while cell cycle arrest was not observed (data not shown). Similar effects were observed when cells were treated with GSK343 (Figure 3D). Induction of apoptosis by UNC1999 was further supported by the cleavage of caspase-8, 9 and 3 (Figure S3B). Furthermore, UNC1999 significantly inhibited colony formation by the MM cell lines INA-6 and RPMI-8226 (Figure 3E). Moreover, we examined the effect of UNC1999 on primary CD138+ plasma cells purified from 12 newly-diagnosed MM patients (Figure 3F). Purified tumor samples from 9/12 patients responded to 72 hours treatment of 4 μM UNC1999 with 40% to 75% decrease in the number of viable CD138+ myeloma cells as compared with UNC2400 treatment.

### UNC1999 reduces the global levels of H3K27 methylation marks

UNC1999 is an orally bioavailable specific inhibitor of EZH2 and EZH1, the enzymatic subunits of the PRC2 complex that catalyze the formation of H3K27me3 [33]. Western blot analysis revealed that UNC1999, but not the inactive form UNC2400, reduced the global levels of H3K27 methylation marks in INA-6 and RPMI-8226 cell lines (Figure 3G). This downregulation in H3K27 methylation was accompanied by an increase in transcriptionally permissive H3K27 acetylation. Treatment with UNC1999 did not affect the global levels of H3K36me2, H3K4me3 and H3K9me2 marks (Figure 3G). Similar to UNC1999, GSK343 treatment led to downregulation of H3K27me3 levels in MM cell lines (Figure S4).

### EZH2 inhibition by UNC1999 induces pro-apoptotic gene expression and downregulates oncogenes in MM

**Figure 4:**
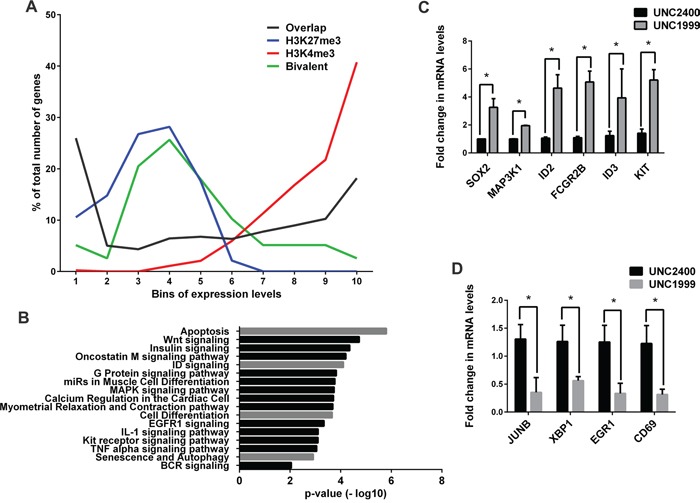
UNC1999 gene expression signature in MM **A.** A correlation between INA-6 RNA expression array control (DMSO) treatment, MM patient RNA-Seq and MM patient ChIP-Seq data sets. P-value was calculated using Chi-square test (p <0.0001). **B.** Biological pathways enriched among the UNC1999 upregulated gene signature. **C-D.** RT-qPCR validation of selected genes among the upregulated **C.** and downregulated **D.** transcripts. Error bars represent the standard deviation of the three independent biological experiments. P-values were calculated using two-tailed student t-test (p * < 0.05).

To dissect the underlying molecular mechanisms for the UNC1999 induced anti-myeloma effect, we performed gene expression microarray analysis on the INA-6 cell line after treatment with 2 μM UNC1999 or UNC2400 for 72 hours. In order to investigate how well the corresponding gene expression pattern of INA-6 cells represented that of the MM patient cells used for the ChIP-seq analysis, we compared the expression data sets of UNC2400-treated INA-6 and patient RNA-seq. We divided the genes from both datasets into ten bins based on their expression levels with the first bin having the least expressed genes and the tenth bin having the most highly expressed genes. This showed that while the genes with moderate expression levels were randomly distributed across middle bins (fourth to seventh), there was selective enrichment of common genes in the bin pairs representing lowest (1246 genes in first bin pair) or highest (871 genes in the last bin pair) expression levels (Figure 4A, black line). We then analyzed if the H3K4me3 and H3K27me3 marks obtained from MM patients correlated with the silenced or highly-transcribed genes common to INA-6 and MM datasets. Overall, we observed significant correlation (p <0.0001) between the transcriptional activity and the presence of the chromatin marks. The genes commonly silenced (first bin pair) and commonly highly-expressed (last bin pair) associated with H3K27me3 (blue line) and H3K4me3 (red line) marks respectively. The bivalent histone marks (green line) were enriched in the intermediate bin pairs with no selective depletion in highly expressed or silenced genes (Figure 4A).

UNC1999 treatment of INA-6 significantly (p=0.02 and fold change of ≥ and ≤ 1.5) altered the expression of 114 transcripts when compared with UNC2400, of which 72 genes were upregulated and 42 genes were downregulated (Table S8). Pathway analysis of the upregulated genes revealed that EZH2 inhibition using UNC1999 significantly upregulated transcripts related to apoptosis, Wnt, ID, MAPK, insulin signaling pathways and cellular differentiation (Figure 4B). The downregulated genes were mainly involved in metabolic activity. Of particular importance is that the inhibition of EZH2 downregulated genes, such as JUNB, CD69 and XBP1, which are known to have oncogenic properties in MM [37–42] (Table S8). The changes in gene expression upon UNC1999 treatment were verified for selected genes by RT-qPCR (Figure 4C and 4D and Figure S6B) and bioinformatics analysis (Figure S6A).

In addition, the upregulated gene signature upon UNC1999 treatment was compared to the unique bivalent and H3K27me3 targets common among the MM patients included in this study. Notably, overlap was only observed among the bivalent genes (p <0.001) (Figure S5A). To examine the chromatin state of the overlapping genes in the INA-6 cell line, we performed ChIP-qPCR analysis. We found EZH2 recruitment at all loci of interest as well as comparable enrichment for both H3K27me3 and H3K4me3 for ID2, FAM65B and SORL1, whereas a higher enrichment for H3K4me3 than for H3K27me3 was observed at SOX2 (Figure S5B).

## DISCUSSION

Development of targeted therapy for multiple myeloma (MM) has been hampered by the complex biology and extensive heterogeneity of the disease [2, 4–7, 43, 44]. Using integrative genomics, we have previously identified Polycomb gene targeting as a common denominator of underexpressed genes in the malignant plasma cell [15]. In the current study, we show for the first time the genome-wide enrichment of H3K27me3 and H3K4me3 in freshly isolated CD138+ cells from newly-diagnosed MM patients. We observed a strong correlation between the H3K27me3 mark and loss of transcriptional activity, whereas H3K4me3 was associated with highly transcriptionally active genes. Overall, the bivalent genes appeared to be transcriptionally silent; however, some transcriptional activity was evident when compared to the H3K27me3-enriched genes. Consistent with this, the bivalent genes clustered together with the H3K27me3 genes when performing hierarchical clustering based on gene ontology (GO) analysis of biological processes. Further supporting this notion, the GO analysis revealed that the myeloma H3K27me3 and bivalent genes were enriched within *development*, *differentiation*, *morphogenesis* and *metabolism* categories (data not shown). This classification is in line with the nature of Polycomb targets and their function in development and differentiation [17].

To distinguish between developmental regulators targeted by Polycomb during normal plasma cell differentiation and those aberrantly silenced in MM, we filtered out H3K27me3-enriched regions in normal bone marrow plasma cells derived from age-matched healthy donors. To examine the Polycomb target genes unique to MM, the gene lists of H3K27me3-only and bivalent targets were analyzed using Oncomine and found to significantly overlap with previously-defined Polycomb targets in human embryonic fibroblasts [27] and in human embryonic stem cells [28]. Notably, such findings have previously been used to define a stem cell-like phenotype in poorly differentiated cancers and have been linked to tumor aggressiveness [45, 46]. In contrast, the malignant cell of MM represents a post-germinal center, isotype-switched, terminally differentiated long-lived plasma cell [3], which makes this connection to stem cell-like features intriguing and suggests that gene silencing may be an important process during tumor development by possibly preserving proliferation over differentiation. Interestingly, an aberrant epigenetic program persisting through the normal cell differentiation process was implicated in tumor initiation when hematopoietic progenitors were proposed to be cells of origin in MM [47]. At what stage during B cell differentiation the MM-associated Polycomb gene targeting and silencing is established and if this is required for transformation remains to be elucidated.

Upon further analysis using Oncomine, a significant correlation with gene expression data from large MM cohorts was identified. H3K27me3-only targets were significantly enriched among genes underexpressed in MM plasma cells when compared to MGUS and normal bone marrow plasma cells [29, 48, 49]. Notably, the MM H3K27me3-enriched genes significantly overlapped with underexpressed genes in the more aggressive MM ISS stage III [50] as compared with stage I and II [29]. Importantly, we also found that H3K27me3 targets in MM define a set of genes underexpressed in patients with a poor survival as defined by two independent studies [30, 31]. This correlation warrants further investigation on how Polycomb gene targeting associates with the molecular classification of MM [30] and the possibility that aberrant Polycomb targeting may be present among several high-risk molecular subgroups.

Interestingly, we found an increased number of bivalent genes in MM when compared with normal plasma cells, with a small number of overlapping genes. This is reminiscent of recent findings in germinal center derived B-cell lymphoma reporting a disease-specific increase of bivalent domains [51]. When the common MM-bivalent genes were analyzed in the Oncomine database we found that they were enriched among the underexpressed genes in MM cell lines as compared with other cancer cell lines. Furthermore, the MM-bivalent genes were enriched among genes previously found to be reactivated upon knockdown of Polycomb proteins. Notably, when we defined upregulated genes in MM upon pharmacological inhibition of EZH2 and compared those to the common-among-patients H3K27me3-only and bivalent genes, an overlap was only identified between the upregulated genes and the bivalent genes. Upon closer inspection of these genes, we found EZH2 recruitment and comparable levels of H3K27me3 and H3K4me3 at 3/4 genes, which is indicative of bivalency. Taken together, this suggests that bivalent domains play an important role in MM, are poised for expression upon external and internal stimuli and could be amenable to pharmacological manipulation.

It is well established that the PRC2 methyltransferase EZH2 functions as an oncogene in several cancers [20, 21]. EZH2 pathologic activation due to gain-of-function mutations and the associated increase in H3K27me3 at target genes have been clearly demonstrated to be essential for the development of germinal center derived B-cell lymphomas [51–54]. EZH2 inhibition using small molecule inhibitors shows potent anti-tumor effects and has minimal toxic effects *in vivo* [33, 35, 51]. In MM, EZH2 overexpression has previously been reported by us and others [15, 22, 49], whereas recurrent mutations have not been reported. In the current study, we explored whether EZH2 inhibition using the selective inhibitors UNC1999 [33] and GSK343 [34] had anti-myeloma effects. Both inhibitors suppressed the growth of a panel of MM cell lines in a dose- and time-dependent manner by induction of apoptosis, but not through cell cycle arrest. In addition, UNC1999 inhibited colony formation by the INA-6 and RPMI-8226 cell lines. Interestingly, the MM cell line INA-6 was sensitive to EZH2 inhibition in a dose range previously demonstrated for the DLBCL cell line Karpas-422, which harbors a gain-of-function mutation in EZH2. However, all MM cell lines in this study had a wild type EZH2 SET domain. Similarly, Beguelin *et al.* found that sensitivity to EZH2 inhibition was determined by dependence on EZH2 and the germinal center origin of the tumor rather than EZH2 mutational status [51]. Furthermore, the MM cell lines included in this study were representative of the major chromosomal translocations detected in MM patients, and responsiveness to EZH2 inhibition was not found to correlate to any of the genetic abnormalities present in the cell lines. Most importantly, the majority (9/12) of CD138+ myeloma cells derived from MM patients responded to UNC1999 treatment, at a concentration range similar to that used in MM cell lines. Considering this, and the fact that UNC1999, as well as other novel EZH2 inhibitors, are available for use *in vivo* [35, 55], it remains to be evaluated if EZH2 will constitute a novel druggable target with potent anti-myeloma effect in relevant murine models of MM.

We observed a global decrease in the levels of H3K27 methylation marks, accompanied by an increase in H3K27 acetylation as a result of treatment with UNC1999 and GSK343. Other histone marks such as H3K4me3, H3K36me2 and H3K9me2 remained unaltered, indicating the target specificity of both inhibitors in MM. EZH2 inhibition using UNC1999 also reactivated the expression of genes in pathways relevant to cancer such as apoptosis, ID signaling and cellular differentiation. Resistance to apoptosis has been linked to disease pathogenesis and resistance to treatment in MM [56, 57]. The inhibitor of DNA binding 2 (ID2) was significantly upregulated in the INA-6 cell line upon treatment with UNC1999. ID2 is an important mediator of both plasma cell differentiation [58] and induction of apoptosis [59] and the ID signaling pathway has previously been reported to be deregulated in cancer [60, 61]. Moreover, we found the FcγRIIb (FCGR2B) transcript among the significantly upregulated genes. Notably, murine plasma cells that lack FcγRIIb expression are resistant to apoptosis [62].

EZH2 inhibition by UNC1999 decreased the expression of several oncogenes, which have been demonstrated to be involved in MM, most notably JUNB, CD69 and XBP1. JUNB has been recently shown to promote MM cell growth, survival and drug resistance [37]. CD69 is a B cell activation cell surface marker, which is suppressed by BLIMP-1 during normal plasma cell differentiation [38]. However, CD69 is more expressed in MM and MGUS than in normal samples [39] and is suggested to be a potential clinical marker in MM [40]. XBP1 is critical in coordinating normal plasma cell differentiation and survival by regulating the unfolded protein response [41] and is reported to drive MM pathogenesis in mice [42].

In conclusion, we present the first genome-wide enrichment analysis of the Polycomb chromatin mark H3K27me3 associated with gene silencing, as opposed to the activating H3K4me3 mark in MM. We identified that genes enriched for H3K27me3 in MM significantly overlap with underexpressed genes in MM ISS stage III and in MM patients with poor survival. We provide evidence that EZH2 inhibition suppresses viability and colony formation in MM cell lines, reduces survival of primary MM cells and leads to the activation of apoptosis. Collectively, these data suggest that EZH2, the enzymatic subunit of PRC2, is a potential therapeutic target in MM.

## MATERIALS AND METHODS

### Primary cells

Heparinized bone marrow samples were obtained from newly-diagnosed MM patients in accordance with the Declaration of Helsinki and approved by the local ethics committees of Uppsala and Stockholm (Dnr 2004:M-332 and 2010/1478-32) (Tables S1 and S2). Healthy bone marrow samples were obtained from patients undergoing selective hip replacement surgery at Karolinska University Hospital. The study was approved by the regional ethical committee (2012/517-32), and all patients gave informed consent. Subjects were considered hematologically healthy if peripheral blood counts were normal, with no signs of inflammation and no history of use of any drugs known to affect hematopoiesis. During surgery, 5-15 mL bone marrow was removed and placed in RPMI media containing 1% glutamax and 16.7 IU/mL heparin.

Mononuclear cells were separated by Ficoll-Paque™ PLUS density sedimentation (Amersham Biosciences, Little Chalfont, UK) and subjected to CD138 immunomagnetic purification by Whole Blood Column Kit (MACS, Miltenyi Biotec, Paris, France) according to the manufacturer's protocols. Subsequently, the purity of the CD138-enriched fraction was evaluated by May-Grünwald-Giemsa staining and samples with purity above 85% for MM, and above 80% for healthy controls were included in the study (Tables S1 and S2).

### Cell culture

All MM authenticated cell lines [63] and primary cells were maintained in RPMI-1640 AQmediaTM (Sigma) supplemented with 10% FBS (Sigma), 1% GlutaMax™-I (Gibco) and antibiotics (penicillin 100 U/mL and streptomycin 50 mg/mL; Sigma) at 37°C in a humidified 5% CO2 in-air atmosphere. For experiments, exponentially growing cells were seeded at 100 000 cells/mL unless otherwise stated. Cells were incubated overnight before addition of reagents. Medium and reagents were refreshed at day 3. Primary cells were maintained at 1 × 10^6^ cells/mL in the presence of IL-6.

### Cell viability assay

MM cell lines were treated with a range of concentrations of UNC1999 (or GSK343) or with DMSO for 6 days, while primary cells were treated with 4 μM UNC1999 or UNC2400 for 72h. On the day of analysis, cells were seeded in triplicate wells in 96-well flat-bottom plates for cell lines and U-bottom plates for patient samples. Cell viability was assessed using Resazurin assay using AlamarBlue (Sigma-Aldrich) as previously described [15]. Only samples where the control treatment reached fluorescence levels of at least 5 × the background for cell lines and at least 2 × the background for patient samples were included in the analysis.

### Apoptosis assay

Cells were cultured for 72 hours in the absence (DMSO control) or presence of EZH2 inhibitor (2 μM UNC1999 or GSK343). Apoptosis was quantified by Annexin V (AV)-fluorescein isothocyanate (FITC) and PI staining using TACS Annexin V-FITC Apoptosis Kit (R&D Systems, Gaithersburg, MD, USA). Samples were treated according to manufacturer's recommendations and analyzed by flow cytometry (FACScan), presenting apoptotic cells as Annexin V-positive/PI-negative cells and necrotic cells as Annexin V-positive/PI-positive cells.

### Colony forming cell assay

A hematopoietic colony-forming cell assay was performed using the CytoSelect 96-well Hematopoietic Colony Forming Cell Assay kit according to the manufacturer's protocol (Cell Biolabs). Briefly, 100 000 - 500 000 cells/mL were plated in CytoSelect methylcellulose medium in 96-well culture plates in the presence of 2 μM UNC1999 or UNC2400. At 7 days, colonies were quantified using a fluorescence plate reader (485/520 nm filter set) using Wallac VICTOR Multilabel Counter (Wallac, Turku, Finland).

### Protein extraction and western blot

Cells were seeded, treated with DMSO, UNC2400 or a range of concentrations of UNC1999 and harvested at the indicated time-points. For global analysis of chromatin modifications by western blot, histone proteins were extracted using the Episeeker histone extraction kit (abcam, Ab113476) following the manufacturer's procedure. For caspases activation analysis, cells were collected by centrifugation at 1500 *rpm* for 5 minutes, washed with ice-cold PBS and lysed in buffer containing 1% NP40, 0.1 M Tris-HCl, 0.15 M NaCl, 5 mM EDTA and protease inhibitors 1 mM ZnCl2, 50 mM Na2MoO4, 10 mM NaF, 0.1 mM NaVO3, 1 mM PMSF, 1 mM DTT, 1× complete EDTA-free protease inhibitor (Roche, Mannheim, Germany). Cell lysates were collected by centrifugation at 12 000 rpm for 10 minutes. Proteins were fractionated on NuPAGE^®^ Novex^®^ Bis-Tris gels (Invitrogen, Carlsbad, CA) and transferred onto a nitrocellulose membrane using the iBlot^®^ system (Invitrogen). The membrane was blocked in 5% non-fat dry milk in TBS (10 mM Tris-HCl, pH 7.7, 150 mM NaCl) with 0.1% Tween 20 (TTBS) at room temperature for 1 hour, incubated with the indicated primary antibodies overnight at 4°C, washed in TTBS and incubated for 1 hour at room temperature with the corresponding secondary HRP-conjugated antibodies (Amersham Biosciences) in 5% non-fat dry milk in TTBS. Antibodies used are listed in Table S3.

### RNA extraction, RNA array, cDNA synthesis and quantitative real time qRT-PCR

RNA extraction for RNA-seq was done from 1 × 10^6^ CD138-selected cells from patient number 3 using the RNeasy Mini Kit (Qiagen) according to the manufacturer's protocol. Similarly, for the INA-6 cell line microarray the RNA was extracted from the cells using RNASpin mini (GE Healthcare) after 72h of treatment with 2 μM UNC1999. The labeling of the RNA for microarray and hybridization were done according the Affymetrix manufacturer's protocol (Affymetrix). The data was analyzed using GeneSpring 13 software using quantile normalization and ExonRMA 16 was used for summarization. The differentially regulated genes were obtained using moderated t-test with the p < 0.02 and fold change 1.5. cDNA preparations were done using Invitrogen kit as per the manufacturer's protocol. For qPCR TaqMan^®^ (Invitrogen) and SYBR^®^ Green (Invitrogen) chemistry was used according the manufacturers’ protocols (Table S4).

### Chromatin immunoprecipitation

ChIP was performed on either freshly isolated CD138-selected cells or on the exponentially growing INA-6 cell line by using a modified version of the OneDay ChIP kit (Diagenode, Liège, Belgium) as previously described [15]. Chromatin was sonicated (30 sec ON/30 sec OFF) at ultrasonic wave output power 320 W in Bioruptor^®^ (Diagenode, Liège, Belgium) until DNA fragments accumulated within 200 to 500 bp size. The immunoprecipitation was performed using 10 μg of chromatin per 1 μg antibody. A list of the antibodies used in this study is found in supplementary data (Table S3). DNA was purified using phenol/chloroform precipitation when samples were submitted for ChIP sequencing and by following the OneDay ChIP kit manufacturer's protocol.

### Sequencing, library preparation and reads alignment

SOLiD™ (Life Technologies) platform was used for sequencing, the library preparation was done according to SOLiD™ protocol for both ChIP-seq and RNA-seq libraries and raw reads were obtained in color space. ChIP-seq reads were aligned using BioScope (Life technologies, CA) while input and RNA-seq reads were aligned using LifeScope (Life Technologies, CA). The reads (Table S9) were mapped uniquely to the reference human genome library hg19. All data have been submitted to the Gene Expression Omnibus (GEO) repository under the accession GSE53215.

### Peak calling

MACS1.4.1 software [64] with parameters, band width 300 (default), mappable genome size 2.70e+09, p-value cutoff 1.00e-05 (default) was used for peak calling. Three criteria were used to call the peaks; first calling the peaks in the patients the bam file for H3K27me3 was used as a test and H3K4me3 file of the same patient as control and vice-versa. Second, an input bam file from one of the patients was used as a control for calling patient H3K27me3 and H3K4me3 peaks and overlapping peaks from these were designated as bivalent. Third, for calling the patients unique peaks for H3K27me3 and H3K4me3 the patients’ bam files were used as test and normal plasma cell bam files of H3K27me3 and H3K4me3 as control. Normal plasma cell bivalent genes were defined by using normal plasma cell input in the similar manner described for the patient bivalent. Peaks were annotated around the transcriptional start site (TSS) using PeakAnalyzer software [65]. The annotation file used was Homo_sapiens.GRCh37.58.gtf.gz. Genes were enriched for H3K27me3 if a peak was detected up to 2.5 kb upstream of the TSS, through the whole gene body and up to 2.5 kb beyond the 3′ end of the gene; genes were enriched for H3K4me3 if a peak was up to 2.5 kb up and downstream of the TSS; and for bivalent peaks 5 kb up and downstream of the TSS. The bivalent genes were generated by intersecting the overlapping regions in the peak bed files, created by the second criteria as mentioned above, using the BEDTools software [66].

### Annotations

Functional annotation was performed using GOStats software (Beissbarth and Speed 2004). For each patient the genes carrying the unique H3K27me3 or H3K4me3 chromatin mark were selected based on the criteria described above, then bivalent genes of each patient were removed and the file was annotated for biological functions. Hierarchical clustering was done using R on the P-values obtained by GOStats functional annotation. Footprints of the histone marks surrounding the peaks were created using the SICTIN software and custom R-scripts (Enroth, Andersson et al. 2010).

### Statistical analysis

Student t-test, Wilcoxon test, chi-square test and moderated t-test were calculated using R and GraphPad Prism.

## SUPPLEMENTARY DATA FIGURES AND TABLES










