# Supplementary material for: Genome-wide profiling of histone H3 lysine 27 and lysine 4 trimethylation in multiple myeloma reveals the importance of Polycomb gene targeting and highlights EZH2 as a potential therapeutic target
Source: Oncotarget. 2016 Jan 7;7(6):6809–23. doi: 10.18632/oncotarget.6843 (PMC4872750; doi:10.18632/oncotarget.6843)
Supplement: Supplementary file 1 [file oncotarget-07-6809-s001.pdf]

SUPPLEMENTARY DATA

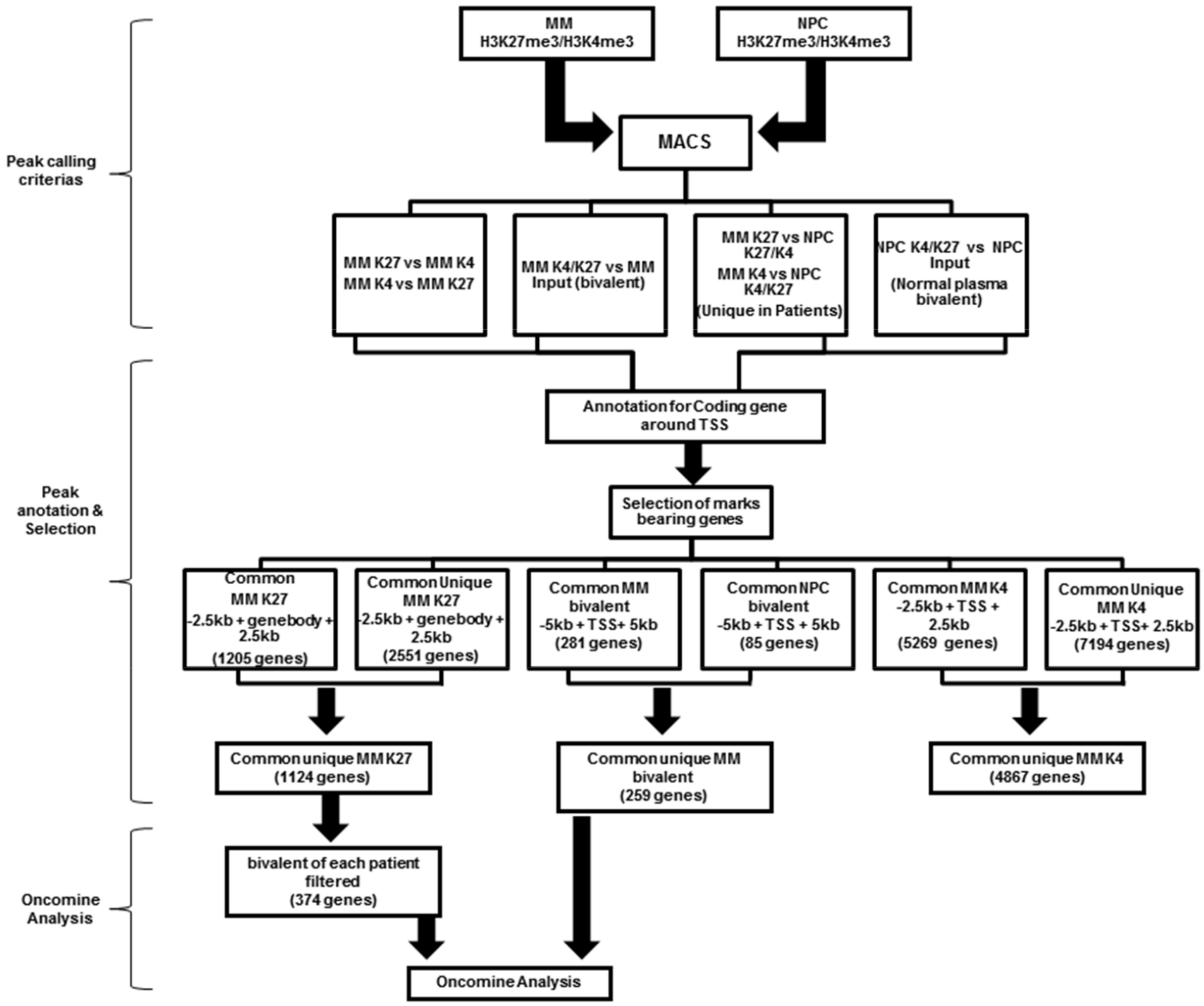

Supplementary Figure S1: ChIP-seq work flow in this paper.

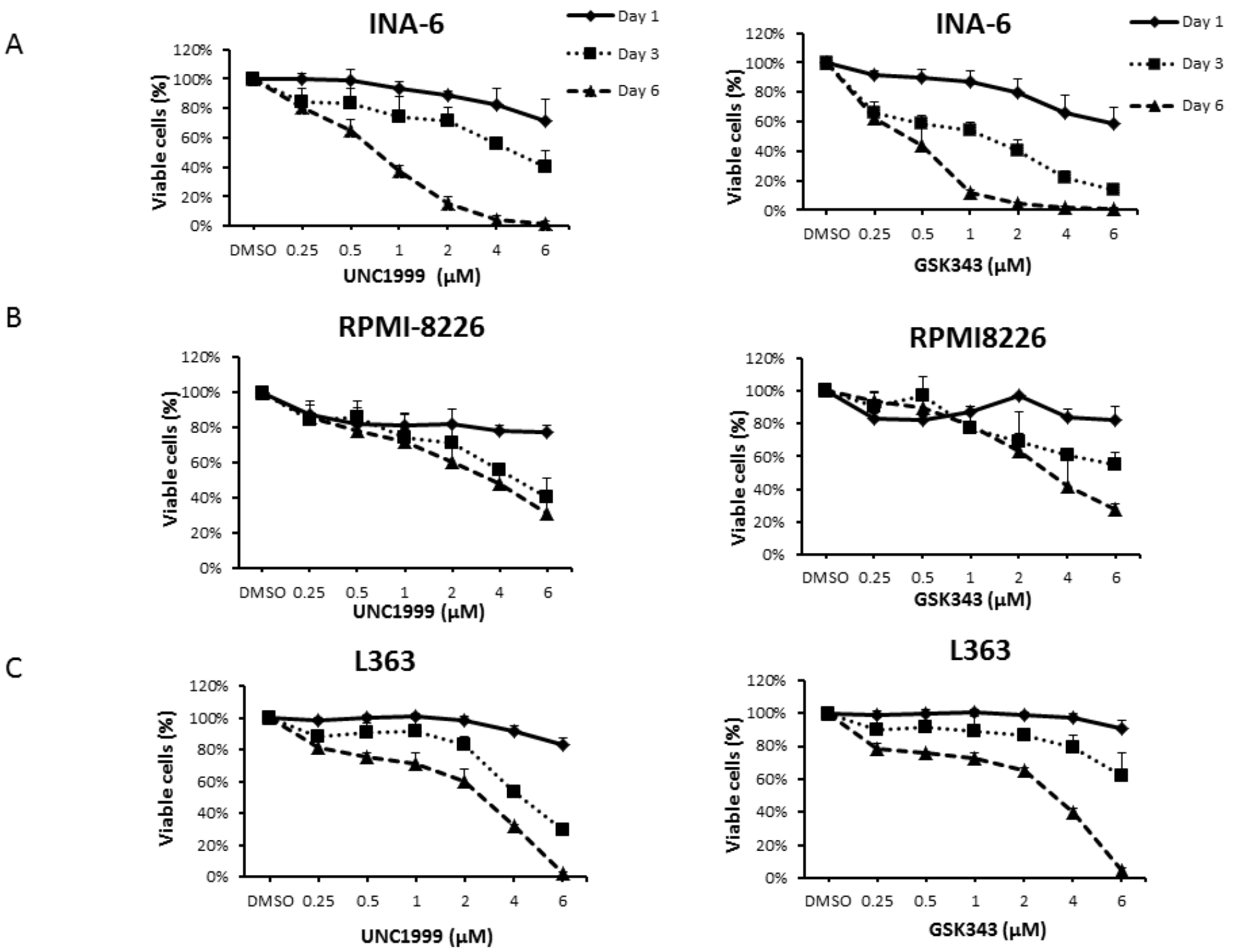

Supplementary Figure S2: Pharmacological inhibition of EZH2 suppresses the growth of MM cell lines in a dose- and time-dependent manner. EZH2 inhibition using UNC1999 (left) and GSK343 (right) reduced the growth of the MM cell lines; INA-6 **A.** RPMI-8226 **B.** and L363 **C.** in a concentration and time dependent manner. DMSO was used as control treatment and cell viability was measured using AlamarBlue assay at days 1, 3 and 6 posttreatment. Error bars represent the standard deviation of three independent experiments.

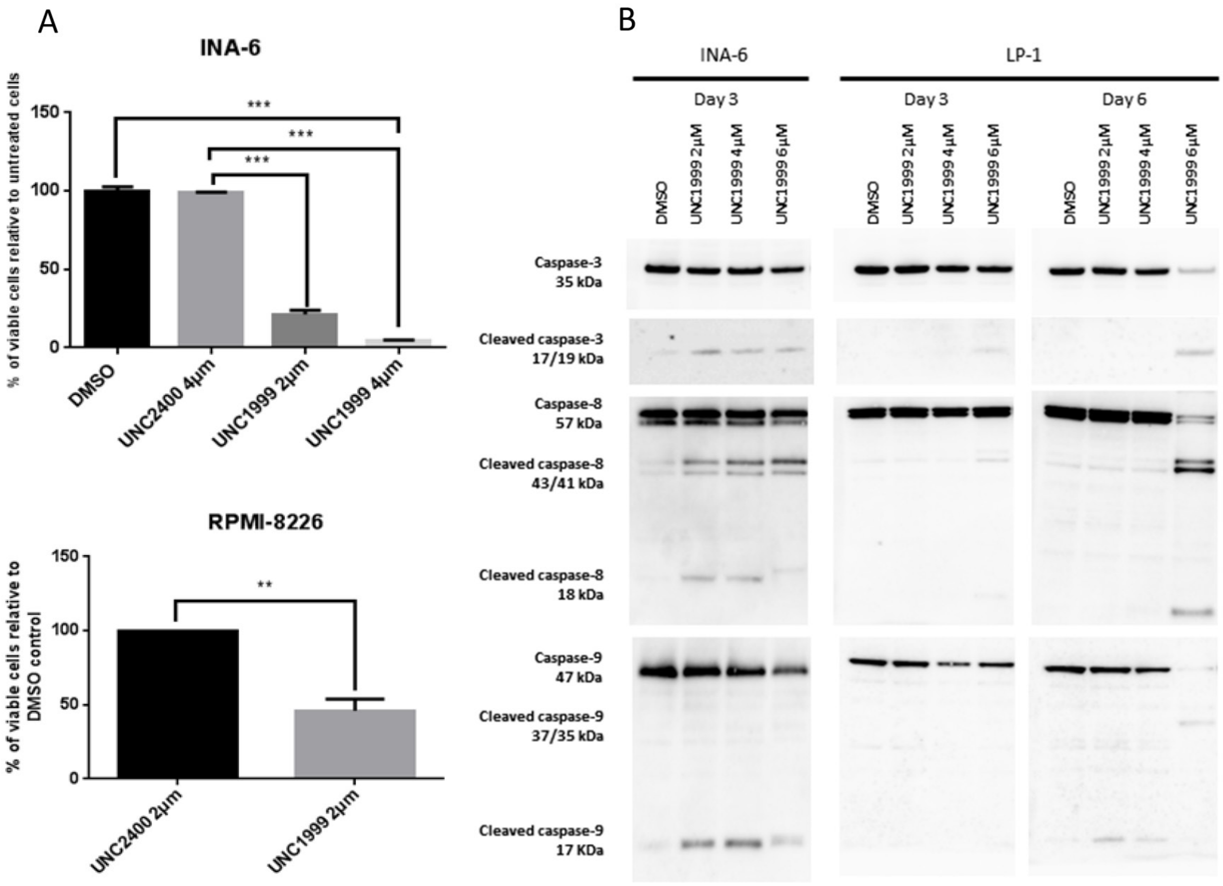

Supplementary Figure S3: UNC1999 induces apoptosis in MM cell lines. A. The inactive chemical analogue UNC2400 did not affect the viability of the MM cell lines, INA-6 and RPMI-8226. Cell viability was analyzed using AlamarBlue assay 6 days posttreatment. **B.** UNC1999 induced apoptosis in the MM cell lines, INA-6 and LP-1 as evidenced by the accumulation of cleaved caspases. The error bars represent the standard deviation of three independent biological experiments and the western blot is a representative of three independent experiments. P-values were calculated using the two-tailed student t-test, p: **<0.01; ***<0.001.

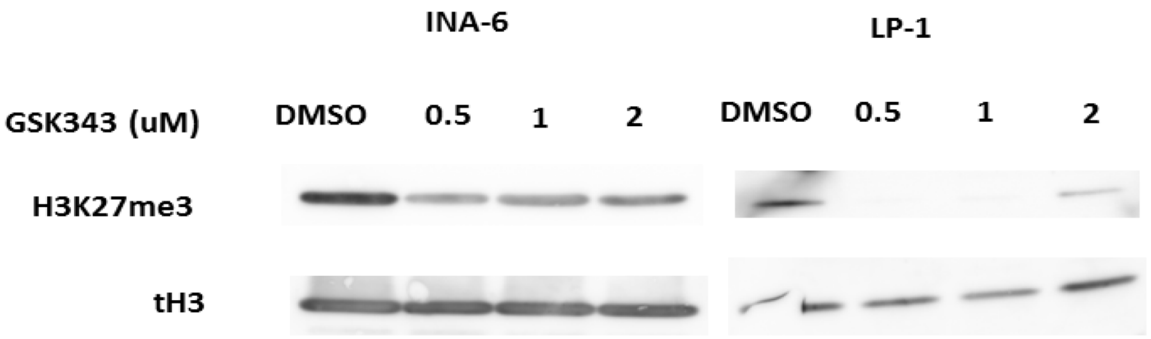

Supplementary Figure S4: GSK343 downregulates the global levels of H3K27me3 mark in MM cell lines. Cells were treated with GSK343 for 72 hours.

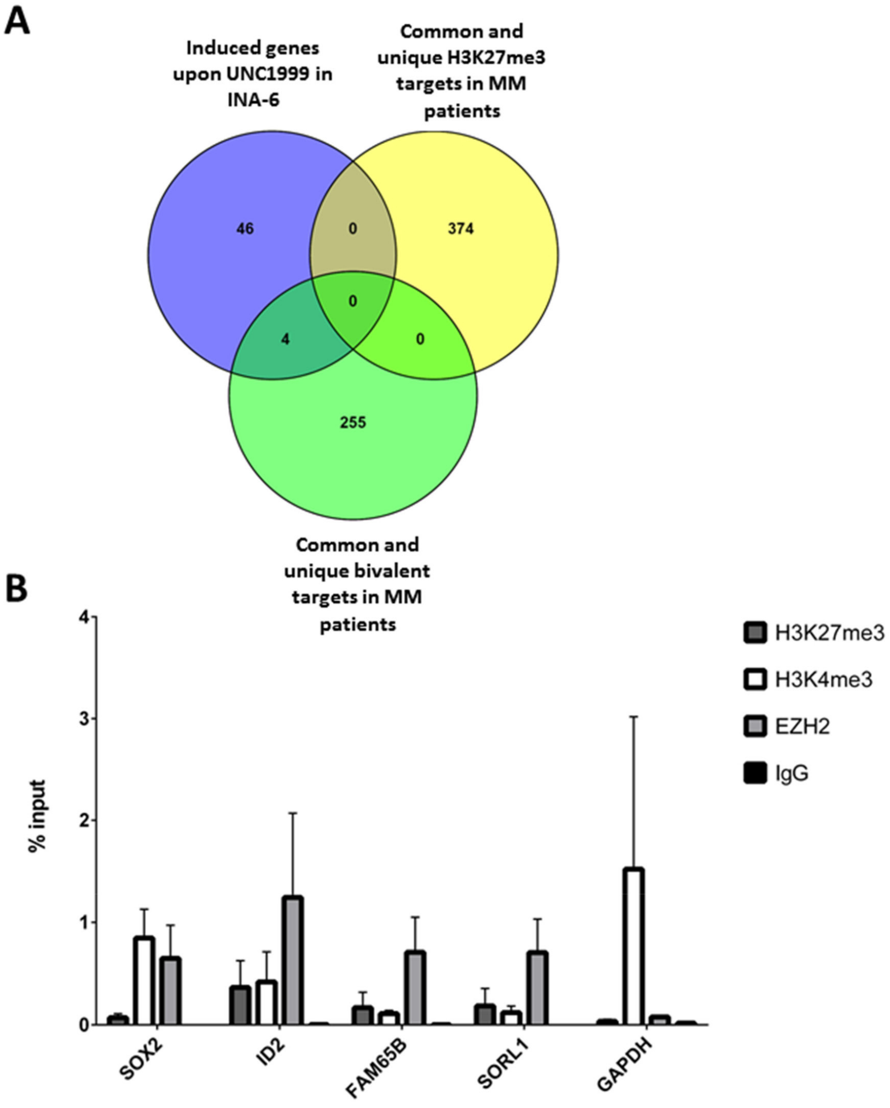

Supplementary Figure S5: UNC1999 reactivates the expression of bivalent genes in MM patients. A. An overlap between the UNC1999-mediated upregulated genes in INA-6 and the MM patients unique H3K27me3 and bivalent genes (p <0.001). Intersection was generated by using Venny 2.0.2 (Oliveros 2007-2015). **B.** ChIP-qPCR validation of bivalency for the MM reactivated genes in INA-6. ChIP analysis was carried out on native INA-6 cell line to study the enrichment of H3K4me3, H3K27me3 and EZH2 at the promoter regions of the reactived genes. GAPDH gene is shown as a control for active genes to discriminate between bivalency and transcriptionally active genes. Error bars represent the standard deviation of the three independent biological experiments.

A

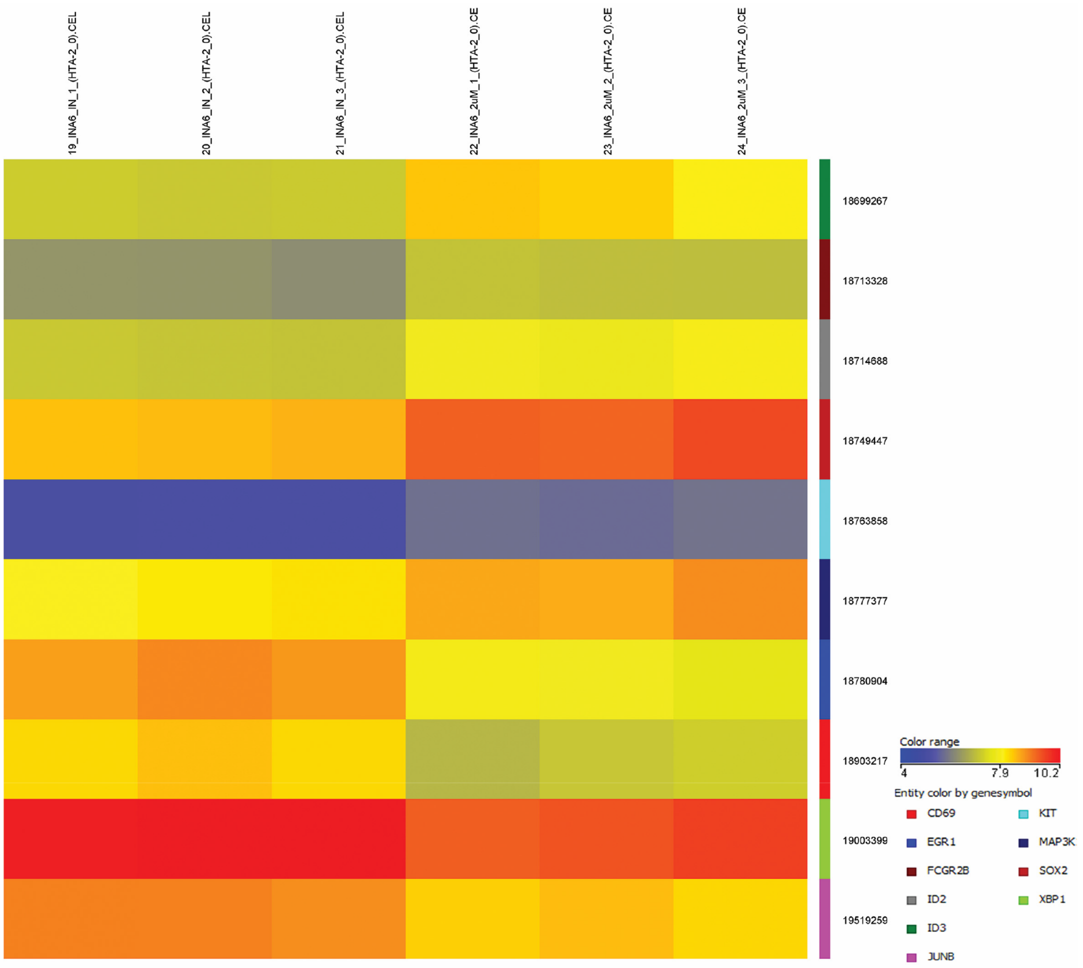

B

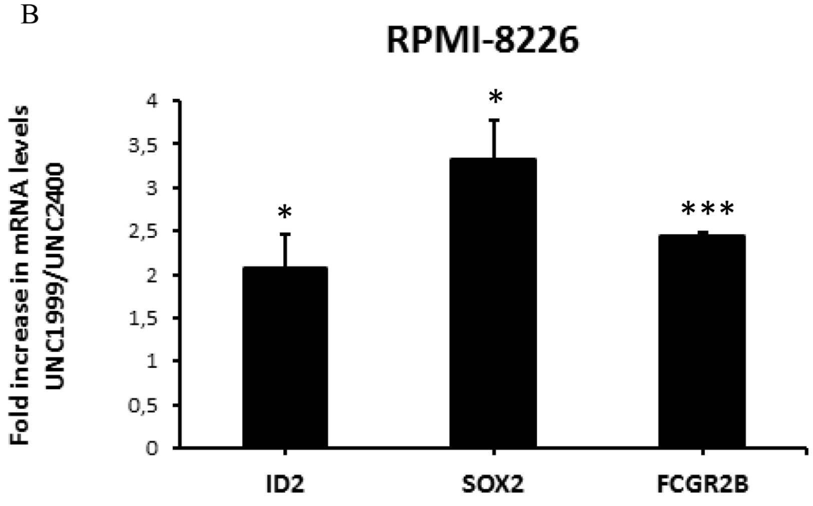

Supplementary Figure S6: A. Heatmap of differentially regulated genes by treatment of UNC1999 as compared to UNC2400 in INA-6. **B.** RT-qPCR confirmation of upregulation of apoptotic genes at 72 hours posttreatment with 2µM UNC1999 in RPMI-8226 cell line. Error bars represent the standard deviation of three independent biological experiments. P-values were calculated using the two-tailed student t-test, p: *<0.05; ***<0.001.

Supplementary Table S1: Disease status (2003) of patients from which plasma cells were isolated and used for ChIP-seq and cell purity after CD138$^+$ selection

Patient	Disease stage / ISS stage	Ig Isotype	% plasma cells
1	multiple myeloma / ISS stage II	G/λ	95
2	multiple myeloma / ISS stage II	G/λ	98
3	multiple myeloma / ISS stage II	G/λ	86
4	multiple myeloma / ISS stage II	G/λ	98

ISS: international staging system for multiple myeloma (Greipp, San Miguel et al. 2005)

Supplementary Table S2: Disease status (2003) of patients from which plasma cells were isolated and used for cell viability screen and cell purity after $CD138^+$ selection

Patient	Disease stage / ISS stage	Ig Isotype	% plasma cells
1	MGUS	G/κ	96
2	multiple myeloma / ISS stage III	G/κ	86
3	multiple myeloma / ISS stage II	A/λ	99
4	multiple myeloma / ISS stage II	A/κ	99
5	multiple myeloma / ISS stage II	G/κ	92
6	multiple myeloma / ISS stage II	A/κ	90
7	smoldering multiple myeloma / ISS stage I	Bence-Jones λ	97
8	multiple myeloma / ISS stage III	G/κ	98
9	multiple myeloma / ISS stage II	G/λ	92
10	smoldering multiple myeloma / ISS stage I	G/κ	87
11	multiple myeloma / ISS stage II	A/κ	96
12	smoldering multiple myeloma / ISS stage I	G/λ	97

ISS: international staging system for multiple myeloma (Greipp, San Miguel et al. 2005). MGUS: monoclonal gammopathy of undetermined significance

Supplementary Table S3: A list of antibodies used in this study

Antibody	Provider / catalog number	Application
Caspase-3	Cell Signaling / 9668	WB
Cleaved caspase-3	Cell Signaling / 9661	WB
Caspase-8	Cell Signaling / 9746	WB
Caspase-9	Cell Signaling / 9502	WB
EZH2	Millipore / CS203195	ChIP
H3K27me3	Millipore / 07-449	ChIP and WB
H3K27me2	Millipore / 07-452	WB
H3K27me1	Millipore / 07-448	WB
H3K27ac	Millipore / 07-360	ChIP and WB
H3K4me3	Millipore / 07-473	ChIP and WB
H3K36me2	Cell Signaling / 2901S	WB
H3K9me2	Cell Signaling / 2753S	WB
H3	Abcam / Ab1791	ChIP and WB
IgG negative control	Diagenode / OneDay ChIP Kit	ChIP

Supplementary Table S4: Primers used for qPCR

Gene	Forward	Reverse	Chemistry
SOX2	TaqMan® probe (Invitrogen)		TaqMan®
MAP3K1	TaqMan® probe (Invitrogen)		TaqMan®
ID2	TaqMan® probe (Invitrogen)		TaqMan®
FCGR2B	TaqMan® probe (Invitrogen)		TaqMan®
KIT	CCACACCCTGTTCACTCCTT	TTCTGGGAAACTCCCATTTG	SYBR® Green
ID3	TGTAGCGGGACTTCTTTTGG	CAGTGGTTCATGTCGTCCAG	SYBR® Green
SETBP1	CAGTTGGCCTTGAAACTGGT	GCATGGTGCTAGGTTTTGGT	SYBR® Green
SP100	AACAAGAACCCGTGGAGTTG	AATACGGTTCTGAGGCGAAA	SYBR® Green
JUNB	GGACGATCTGCACAAGATGA	AGGTAGCTGATGGTGGTCGT	SYBR® Green
XBP1	TCACCCCTCCAGAACATCTC	ACAGAGAAAGGGAGGCTGGT	SYBR® Green
EGR1	CCGCAGAGTCTTTTCCTGAC	TGGGTTGGTCATGCTCACTA	SYBR® Green
CD69	AGTCCCCATTTCTCAACACG	GTAGCCAACCCAGTCCTCAG	SYBR® Green
SOX2	TGGAAATAACTTAAGGAAAGTCTGC	CTTCCCATAATCACTCCCCCG	SYBR® Green
ID2	GGCGGCCCAAGATTGTTTTC	CCGATTTGTGGCTGCGTTAG	SYBR® Green
FAM65B	TCAGACTTACCTGTGTTTCTCAGT	GGTTTGTAATGTTGGCGGAGG	SYBR® Green
SORL1	ACCACACGAACTTCACCATTCT	TGGACTTTAATCCAGACTGAAGAA	SYBR® Green

Supplementary Table S5: List of the common H3K27me3, bivalent and H3K4me3 targets in MM patients

See Supplementary File S5

Supplementary Table S6: The number of peaks and genes for each patient, and their contribution percent to the common list of peaks and genes used for further analysis

	Patient 1 Peaks/Genes	Patient 2 Peaks/Genes	Patient 3 Peaks/Genes	Patient 4 Peaks/Genes	Common peaks	Common Genes
H3K27me3	38195/3527 (17.4/34.2)%	107421/4757 (6.2/25.3)%	81206/4990 (8.2/24.1)%	55044/4907 (12.1/24.5)%	6660	1205
H3K4me3	48252/10178 (20.1/51.8)%	63524/12270 (15.3/42.9)%	55727/1055 (17.4/49.9)%	13306/5655 (73.0/93.2)%	9716	5269
Bivalent	8216/2145 (10.7/13.1)%	10534/2934 (8.2/9.6)%	9310/2788 (9.5/10.1)%	7550/1813 (11.7/15.5)%	882	281

Genes were annotated based on the proximity of TSS selection criteria

Supplementary Table S7: List of the unique H3K7me3 and bivalent targets in MM patients that used in Oncomine search

See Supplementary File S7

Supplementary Table S8: List of significantly UNC1999 regulated genes

See Supplementary File S8

Supplementary Table S9: List of sequencing reads

See Supplementary File S9

REFERENCES

1. (2003). "Criteria for the classification of monoclonal gammopathies, multiple myeloma and related disorders: a report of the International Myeloma Working Group." Br J Haematol 121: 749-757.
2. Greipp, P. R., J. San Miguel, et al. (2005). "International staging system for multiple myeloma." J Clin Oncol 23: 3412-3420.
3. Oliveros, J. C. (2007-2015). Venny. An interactive tool for comparing lists with Venn's diagrams.
